# Fundamental Principles of Bioethics and Their Implementation in the Paediatric Intensive Care Unit: Clinical Complexities and the Imperative for Structured Ethics Education

**DOI:** 10.7759/cureus.108589

**Published:** 2026-05-10

**Authors:** Kalliopi Straka, Eleni Christakou

**Affiliations:** 1 Pediatric Intensive Care Unit, National and Kapodistrian University of Athens, Athens, GRC; 2 Pediatric Intensive Care Unit and Pediatric Cardiac Intensive Care Unit, Aghia Sophia Children's Hospital, Athens, GRC; 3 Pediatric Intensive Care Unit, Aghia Sophia Children's Hospital, Athens, GRC

**Keywords:** bioethics, bioethics education, core principles, medical ethics, picu, training

## Abstract

Paediatric intensive care units (PICUs) are highly complicated and challenging clinical environments where life-or-death decisions must be made urgently, often under conditions of uncertainty and significant emotional pressure. This review examines the practical application of the four fundamental principles of bioethics - autonomy, beneficence, non-maleficence and justice - in PICUs, with a particular focus on the Greek healthcare context. Children’s autonomy is inherently limited, necessitating parental or guardian involvement, yet conflicts often arise when adolescents express preferences that diverge from those of their families. Clinicians must also balance beneficence and non-maleficence in high-risk or emergency interventions, frequently with incomplete information, especially when it comes to complex clinical cases or in children with many comorbidities or congenital health issues. Justice is challenged by scarce resources, compelling clinicians to make ethically correct decisions regarding the allocation of beds or equipment or various types of treatment without standardised institutional guidance or appropriate protocols. In Greece, structured ethics education and formal ethics committees are limited, contributing to variability in practice and an increase in moral distress among healthcare professionals. Given these circumstances, it is considered pivotal to enhance ethics education through curricula, practical workshops and training in communication and to improve the support from each institution in order to strengthen clinicians’ ethical competence, gain public trust and, most importantly, improve patient care. The integration of international guidelines and the provision of psychological support are essential to promote sustainable and ethically correct practice in PICUs.

## Introduction and background

Paediatric intensive care units (PICUs) represent one of the most ethically complex environments in modern medicine, where clinical decisions are frequently made under conditions of uncertainty, urgency, and significant emotional burden for patients, families, and healthcare professionals alike [1,2]. Critically ill children constitute a particularly vulnerable population due not only to the severity of their medical conditions but also to their developmental stage, limited communication abilities, and reduced capacity for autonomous decision-making [3-6]. Consequently, parents or legal guardians are generally required to act as surrogate decision-makers, with the primary responsibility of safeguarding the child’s welfare and best interests.

Ethical challenges emerge when disagreements arise between healthcare professionals and families regarding appropriate treatment goals, or when adolescents capable of understanding their condition express preferences that conflict with parental decisions. In such circumstances, clinicians must carefully balance respect for emerging autonomy, parental authority, and the obligation to act in the child’s best interests [6,7].

The four fundamental principles of bioethics - autonomy, beneficence, non-maleficence, and justice - provide the foundation for ethical reasoning in clinical practice [1]. However, their application in pediatric critical care is often complex and context-dependent. Decisions regarding life-sustaining treatment, experimental therapies, end-of-life care, and resource allocation frequently involve tensions between prolonging survival and preventing suffering. These challenges are further intensified by prognostic uncertainty, rapidly evolving clinical conditions, limited institutional guidance, and the emotional intensity inherent in pediatric critical illness.

Ethical practice in PICUs is additionally influenced by institutional culture, available resources, legal frameworks, and access to structured ethical support. In Greece, these difficulties are compounded by the limited availability of hospital ethics committees, insufficiently developed legal guidance regarding pediatric end-of-life care, and the lack of systematic bioethics education within undergraduate and postgraduate medical training [5,8-10]. As a result, clinicians may experience uncertainty, moral distress, and ethical conflict, particularly when they perceive constraints in acting according to their professional and ethical responsibilities.

These challenges highlight the need to strengthen ethical competence and institutional support within Greek PICUs. Integrating bioethics education into medical and nursing curricula, promoting interdisciplinary ethical deliberation, establishing accessible ethics consultation services, and developing locally adapted clinical guidelines may support more consistent, transparent, and patient- and family-centred decision-making.

This narrative review examines current ethical challenges in pediatric intensive care, with particular emphasis on the Greek healthcare context. It aims to synthesise existing literature, identify gaps in ethics education and institutional support, and explore practical strategies for improving ethical decision-making and compassionate care in PICU practice.

Methods

A narrative literature review was conducted to explore the ethical challenges encountered in PICUs, with particular emphasis on the Greek healthcare setting. A comprehensive search of the literature was performed using electronic databases including PubMed, Scopus, and Google Scholar. Both international and Greek-language sources were considered to ensure a broad and contextually relevant overview of the topic.

The review included peer-reviewed original research articles, review articles, case reports, clinical and ethical guidelines, position statements, educational curricula of professional societies, and relevant national and international legal and regulatory frameworks related to pediatric critical care ethics. Grey literature and policy documents were also reviewed when considered pertinent to clinical practice and ethical governance.

The literature search focused on major ethical issues commonly encountered in daily PICU practice, including ethical decision-making in critically ill children, surrogate and shared decision-making, informed consent, end-of-life care, withholding and withdrawal of life-sustaining treatment, allocation of scarce healthcare resources, and ethics education and training for healthcare professionals. Additional emphasis was placed on the practical application of ethical principles in clinical settings, the identification of institutional and educational gaps, interdisciplinary communication challenges, and clinicians’ experiences within Greek hospitals and PICUs specifically [2,3,5,6,9].

Relevant publications were selected based on their clinical relevance, contribution to ethical discourse, applicability to pediatric intensive care, and relevance to contemporary challenges in both international and Greek clinical practice. The findings were synthesised narratively in order to provide a comprehensive overview of current ethical dilemmas, existing frameworks, and areas requiring further development in pediatric critical care ethics.

## Review

Clinical vignettes anchoring the analysis

The cases described in the following vignettes do not describe real patients, but they are a combination of patients and scenarios coming from our clinical experience.

Vignette A: Continuation Versus Withdrawal of ECMO Amid Disagreement

A nine-year-old child with influenza-associated fulminant myocarditis is admitted in cardiogenic shock. Despite maximal inotropic support and mechanical ventilation, haemodynamic instability progresses, and the child is cannulated for venoarterial extracorporeal membrane oxygenation (VA-ECMO). Over the subsequent week, complications accumulate: progressive acute kidney injury requiring continuous renal replacement therapy, worsening hepatic dysfunction, and recurrent bleeding at cannulation and procedural sites necessitating repeated transfusions. Neurological assessment is constrained by deep sedation; intermittent pupillary asymmetry prompts neuroimaging, which demonstrates diffuse cerebral oedema with changes concerning for hypoxic-ischaemic injury, though prognostication remains uncertain.

By day 8, serial echocardiography and clinical trajectory suggest that myocardial recovery is increasingly unlikely, and the child is not considered a transplant candidate due to evolving multi-organ failure. The multidisciplinary team concludes that continued ECMO is no longer proportionate, sustaining physiological parameters without a reasonable prospect of recovery to a state consistent with the child’s interests, and recommends withdrawal of ECMO with a transition to comfort-focused care. The parents, however, experience the recommendation as morally intolerable: they regard ECMO as “keeping our child alive,” interpret withdrawal as abandonment, and request continuation “until there is no doubt.” Within the team, distress and disagreement emerge regarding framing: some clinicians emphasise the ethical obligation to avoid non-beneficial treatment, while others worry that a firm recommendation will be perceived as rationing or as an erosion of parental authority. Nursing staff report rising moral distress, describing a growing sense of participating in burdensome interventions without a coherent, shared goal. The case illustrates how proportionality, best-interests reasoning, and shared decision-making can become strained when life-sustaining technology outpaces the clinical possibility of recovery and when uncertainty is interpreted differently by families and clinicians.

*Vignette B: ECMO as a Time-Limited Trial (Criteria Not Met, Conflict Escalates*)

A three-month-old infant with congenital diaphragmatic hernia is admitted with refractory respiratory failure and severe pulmonary hypertension. After maximal ventilatory optimisation and inhaled pulmonary vasodilators fail to stabilise oxygenation, the infant is placed on venovenous ECMO. Prior to cannulation, the team proposes ECMO explicitly as a time-limited trial, outlining clinical milestones that would indicate benefit (improvement in oxygenation indices, decreasing pulmonary artery pressures, evidence of lung recruitment) and agreeing to a structured review at seven to 10 days.

By day 10, these milestones have not been achieved. Pulmonary hypertension remains severe, oxygenation shows minimal sustained improvement off high ECMO support, and complications develop, including an intracranial haemorrhage that, while not catastrophic, significantly worsens the infant’s overall prognosis. On review, the team concludes that the trial has not demonstrated benefit and recommends cessation of ECMO with a shift to comfort-focused care.

The parents contest the recommendation and request the extension of the trial, expressing that they did not fully appreciate that ECMO might be stopped in the absence of improvement. They perceive the predetermined milestones as arbitrary and argue that any chance of survival justifies ongoing support. Clinicians experience tension between fidelity to the pre-agreed trial framework and the moral imperative to respond compassionately to parental distress. Across shifts, documentation varies in its account of what was communicated and agreed, with inconsistent phrasing that alternately suggests an open-ended commitment to continued ECMO or a conditional, trial-based approach. This vignette highlights both the ethical value of time-limited trials in contexts of uncertainty and the practical vulnerabilities of such trials when communication, documentation, and intra-team alignment are incomplete.

Vignette C: Limitation of Life-Sustaining Treatment With Parental Disagreement and Moral Distress

A six-year-old child is admitted following a road traffic collision resulting in severe traumatic brain injury. After several days of neurocritical care, neuroimaging demonstrates extensive diffuse axonal injury and refractory cerebral oedema. Neurological examination shows no purposeful responses, and adjunctive investigations are consistent with an extremely poor prognosis for recovery of consciousness. The clinical team concludes that ongoing invasive support is unlikely to achieve outcomes consistent with the child’s interests and recommends transition from aggressive life-sustaining treatment to comfort-focused care.

The parents respond differently. One parent accepts the prognosis and requests that the child not undergo further invasive interventions. The other insists that life support must continue, emphasising hope for an exceptional recovery and viewing withdrawal of ventilation as morally equivalent to causing death. Family meetings become emotionally charged and repetitive, with limited convergence on goals of care. Clinicians struggle to communicate prognostic uncertainty with clarity while maintaining trust; repeated explanations are experienced by the dissenting parent as pressure to consent to limitation. At the bedside, nursing staff report escalating moral distress, describing the difficulty of providing ongoing invasive care in the absence of shared goals and amidst perceived prolongation of dying.

The team considers whether and when ethics consultation should be pursued, how to support parental disagreement without defaulting to indefinite escalation, and how to articulate ethically grounded recommendations while respecting the family’s grief. The vignette underscores the practical and ethical complexity of best-interests determinations in catastrophic neurological injury and the need for structured processes-communication strategies, documentation practices, and team support-to manage conflict, moral distress, and decision-making integrity.

Vignette D: Justice and Resource Limitation (ECMO Candidacy During Scarcity)

During a seasonal surge, the PICU has capacity for only one additional ECMO run. Two critically ill children deteriorate within hours. The first is a previously healthy 12-year-old with fulminant myocarditis and rapidly worsening cardiogenic shock; the team believes there is a plausible prospect of recovery if mechanical circulatory support is instituted promptly. The second is a 10-year-old with complex chronic conditions who develops progressive multi-organ failure following prolonged sepsis; the child meets minimal technical criteria for cannulation but has a substantially lower likelihood of recovery.

Both families advocate strongly for maximal intervention, and both interpret access to ECMO as a test of whether clinicians are “doing everything possible.” Clinicians anticipate that any decision will be morally contested and fear that differential treatment may be perceived as discrimination against disability or chronic illness. Junior staff express uncertainty about how to explain “likelihood of benefit” without reducing the child’s worth to prognosis. Meanwhile, time-sensitive logistics require decisive action.

The leadership team must make and communicate an allocation decision under duress while ensuring transparency, consistency, and documentation. The case illustrates justice considerations at the bedside: the importance of fair processes, consistent criteria, and non-discriminatory reasoning, as well as the ethical and communicative burden placed on clinicians when institutional policies are absent, unclear, or poorly operationalised in frontline practice.

Vignette E: Recurrent Cancer, Adolescent Refusal, and Parental Insistence on Treatment

A 15-year-old adolescent with relapsed acute leukaemia is admitted to the PICU with septic shock and respiratory failure during salvage therapy. The adolescent has experienced multiple lines of treatment, prolonged hospitalisations, and significant treatment-related morbidity. Between episodes of delirium and sedation, the patient is intermittently lucid and expresses refusal of further life-prolonging interventions, stating they do not want additional invasive treatment and prefer comfort. The parents, distressed and determined, insist that “everything must be done,” emphasising that another remission remains possible and that discontinuing intensive treatment would be equivalent to giving up.

Clinicians face layered uncertainties. The adolescent’s decision-making capacity must be assessed in the context of acute illness, fluctuating cognition, fear, and physiological instability. The urgency of care requires sedation, intubation, and invasive monitoring, after which the adolescent’s earlier refusals become a focal point for conflict about ongoing goals of care should deterioration recur. Some clinicians emphasise respect for the adolescent’s emerging autonomy and prior expressed preferences; others highlight the limits of capacity in critical illness and the moral weight of parental responsibility in paediatrics. The team also anticipates that the parents may experience any acknowledgement of the adolescent’s refusal as betrayal or abandonment.

The vignette raises ethical questions about assent and refusal in older adolescents, how to interpret and document preferences expressed during fluctuating lucidity, how to balance parental authority with the patient’s values in the setting of relapse and limited prognosis, and how to conduct shared decision-making conversations without coercion. It also underscores the need for consistent documentation and structured communication, as ambiguity or inconsistency can amplify conflict and erode trust.

Vignette F: Tracheostomy, Long-Term Ventilation, and Contested Quality of Life

A two-year-old child with severe neuromuscular disease is admitted with progressive respiratory failure. Following repeated failed extubations despite optimisation, the clinical team proposes tracheostomy with long-term ventilation. Survival is likely with technology dependence and intensive home-care planning, but prospects for functional improvement are limited, and the child’s trajectory includes recurrent hospitalisations and invasive interventions. The parents are divided: one parent regards tracheostomy as an unacceptable prolongation of suffering and a loss of dignity; the other views it as a pathway to stability and time at home, arguing that the child’s life has intrinsic value irrespective of disability and that the family can adapt.

Within the PICU team, ethical emphasis varies. Some clinicians focus on feasibility, potential benefits, and the family’s capacity to care; others stress cumulative burdens, repeated distressing procedures, and the possibility that ongoing invasive support may no longer serve the child’s interests. Nursing staff report moral distress related both to ongoing invasive care and to the fear of appearing to pressure the family toward limitation. The team considers a time-limited trial of continued ventilation with explicit review points, alongside early involvement of palliative care and ethics support, but remains concerned about how these steps may be interpreted by the family, either as abandonment or as coercive steering.

This vignette highlights the ethical complexity of chronic critical illness in paediatrics: how best-interests reasoning is applied when prognosis involves survival with substantial burdens; how parental authority and family values shape decision-making; how clinicians can use time-limited trials as ethically defensible tools under uncertainty; and how structured ethics education can support consistent framing, conflict-sensitive communication, and transparent documentation.

Across these vignettes, the ethical challenges of paediatric intensive care are revealed not primarily as deficits of goodwill or clinical competence, but as predictable points of collision between foundational principles when decisions must be made under uncertainty, high emotional stakes, and variable interpretations of benefit and burden. ECMO scenarios (Vignettes A-B) foreground proportionality and best-interests reasoning when advanced technologies can sustain physiology without restoring a plausible pathway to recovery, and they illustrate how disagreement escalates when prognostic uncertainty, differing moral meanings of “doing everything,” and inconsistent documentation undermine shared understanding. The end-of-life and neurocritical care scenario (Vignette C) demonstrates how parental disagreement and team moral distress can intensify when goals of care are not stabilised through a structured process. The scarcity scenario (Vignette D) shows that justice considerations and the need for fair, transparent allocation cannot be deferred to abstract policy: they become bedside realities requiring skilled communication and defensible reasoning. The adolescent with relapsed leukaemia (Vignette E) highlights tensions between respect for emerging autonomy, parental authority, and capacity assessment in critical illness, while the tracheostomy and long-term ventilation case (Vignette F) illustrates how chronic critical illness forces repeated, value-laden judgements about acceptable burdens, quality of life, and the moral status of time-limited trials.

Taken together, these cases motivate the paper’s central claim: that principled practice in the PICU requires more than knowledge of ethical concepts-it requires structured, reproducible methods for applying those concepts in real time. In each vignette, the clinical task is simultaneously ethical and communicative: clarifying what counts as benefit, articulating proportionality, negotiating disagreement without coercion, supporting team integrity in the face of moral distress, and documenting reasoning with continuity across shifts and disciplines. The imperative for structured ethics education follows directly: without routine opportunities for multidisciplinary case analysis, deliberate skills training in high-stakes communication, and standardised processes (including clear triggers for ethics involvement and consistent documentation), clinicians are left to improvise under pressure, increasing the risk of unwarranted variation, preventable conflict, and ethically incoherent care. The subsequent sections, therefore, examine how foundational principles can be operationalised through structured education and implementation strategies that support ethically robust decision-making as a matter of everyday PICU practice.

Results: the four fundamental principles

Autonomy

Autonomy in paediatrics, in general, cannot be understood as a fixed or absolute principle. In paediatric care, this principle differs fundamentally from what we mean and apply in adult medical practice. Unlike adult medicine, where autonomy is typically bounded in the patient’s legal authority to express their desires and the patient holds the capacity to make decisions, paediatric autonomy unfolds along a developmental continuum shaped by age, mental development, experience, illness trajectory, family dynamics, and sociocultural context. The cognitive and emotional ability of the child-patient is also of paramount importance in decision-making and perception [4,6]. In the PICU, where decisions are often urgent, technologically complex and emotionally charged, respect for autonomy is necessarily relational and negotiated in a content- dependent manner rather than individually exercised. Because children lack full legal authority to make their own medical decisions, the medical decision-making is delegated to their surrogate decision-makers, most commonly being their biological parents or their legal guardians. This surrogate model is ethically justified by the presumption that parents are the best-positioned persons capable of understanding their child’s needs and advocating for their child’s best interests. However, in practice, surrogate decision-making in the PICU is rarely straightforward. Parents are frequently asked to make decisions under conditions of extreme psychological distress, uncertainty, agony and time pressure, often while dealing with fear, hope, guilt and anticipatory grief. These emotional burdens can significantly shape the parental preferences, sometimes in ways that diverge from what clinicians perceive as the child’s best interests. Sometimes, under these extremely painful emotions, parents demand more aggressive interventions and treatments for their offspring that clinicians regard as disproportionate or harmful [6,7]. Despite its ethical importance, meaningful engagement with children’s perspectives is often underdeveloped in the PICU.

The ethical complexity of autonomy becomes particularly visible in adolescents. Many adolescents in the PICU - especially those who suffer from chronic, relapsing or life-limiting illnesses - possess a level of understanding that allows them to meaningfully engage with information about their prognosis, the treatment burdens and the potential outcomes. They may articulate values related to bodily integrity, dignity, independence or tolerance of suffering. Ethical tension arises when these expressed values conflict with parental wishes or medical recommendations. Yet, the Greek law does not formally recognise decisional autonomy for minors, even when decisional capacity is demonstrably present. As a result, adolescents’ preferences often lack the legal weight, in order to form important decisions about their own welfare.

In Greece, medical decision-making for minors is legally grounded in parental authority and responsibility, as articulated in the Greek Civil Code (Articles 1510-1518), which assigns the parents the duty to care for the person and property of the child and to make decisions in the child’s best interests. While this framework provides legal clarity regarding who holds decisional authority, it offers limited guidance regarding how these crucial decisions should be made in ethically complex clinical contexts, particularly when parental choices conflict with the medical recommendations or the expressed wishes of the sick child.

Consider the case of a 16-year-old adolescent with advanced cystic fibrosis admitted with acute decompensation of chronic respiratory failure. The patient, having experienced repeated traumatic intubations, explicitly refuses invasive mechanical ventilation, expressing a preference for non-invasive support and comfort-focused and palliative care. The parents, however, insist on intubation, framing it as a moral obligation to preserve their life using all the available medical means. Clinicians are placed in a profound ethical dilemma: respecting the adolescent’s emerging autonomy risks contravening parental authority and legal norms, while complying with parental demands may violate the adolescent’s expressed values and expose them to significant suffering even without much benefit. Another example is a 15-year-old patient with leukaemia who may refuse a high-risk chemotherapy regimen after a possible initial treatment failure or cancer remission, while the parents insist on proceeding. In the absence of legal recognition of the mature minor doctrine in Greece, clinicians lack clear legal protection for honouring the adolescent’s refusal, even when are ethically compelling to do so [6,7].

This legal rigidity amplifies even more the moral distress experienced among clinicians, who may feel ethically obligated to engage with adolescents as moral agents, but are legally constrained to disregard their preferences. While Greek medical ethics codes emphasise the respect for the patient’s dignity and their participation in decision-making, they restrict offering actionable guidance in cases of adolescent refusal of treatment. Consequently, clinicians are often forced to navigate these situations informally, often relying on negotiation, persuasion and compromise rather than applying transparent ethical processes.

Respect for autonomy in paediatrics also extends beyond adolescents. Even younger children may express meaningful preferences, fears and values, particularly in relation to invasive or frightening interventions. A child who expresses intense fear of intubation or repeated procedures is communicating experiential knowledge that carries ethical significance. While such expressions do not confer decisional authority, systematically ignoring them risks reducing the child’s personality and making them feel unheard and not respected. The ethical concept of assent attempts to address this gap by encouraging age-appropriate involvement, the inclusion of children in age-appropriate discussions, the acknowledgement of the child’s perspective, desires and minimisation of conflict whenever possible.

Structured frameworks for assessing comprehension and facilitating age-appropriate participation are essential. Including children in discussions about their care has been shown to improve adherence, satisfaction, and trust in healthcare providers [6,7]. Achieving this balance in practice requires deliberate communication strategies and reflective ethical reasoning, skills that remain underdeveloped in PICU clinicians without structured ethics education. However, achieving these practices requires communication skills and ethical sensitivity that are not systematically taught in many Greek PICU training programs.

Respecting emerging autonomy in the PICU, therefore, requires more than theoretical acknowledgement. It demands deliberate institutional commitment to shared decision-making, structured assessment of decisional capacity and reflective ethical practice. Without such support, clinicians may default to either parental authority or medical paternalism, neither of which adequately addresses the moral complexity of paediatric autonomy.

Beneficence and Non-Maleficence

The principles of beneficence and non-maleficence are fundamental to paediatric intensive care, yet their application in everyday clinical practice is dominated by ambiguity and, mainly, by moral uncertainty. Acting according to these principles means that the clinicians have to work towards the patient’s best interests and simultaneously avoid doing more harm [2,3,5]. In the PICU environment, clinicians routinely initiate interventions that are highly invasive, painful and associated with significant short- and long-term morbidity in an urgent and rigorous clinical setting. These interventions are justified by the possibility of benefit - usually regarding the survival of the patient- but the nature, magnitude and likelihood of that benefit are frequently uncertain.

A representative example is the care of neonates suffering from severe hypoxic-ischemic encephalopathy or neonates that are born with complex congenital anomalies requiring multiple surgical interventions and long PICU admissions with many invasive procedures and possible future complications. This aggressive life-sustaining support and treatment may offer survival, but at the cost of probable profound neurological impairment and lifelong dependence on medical technology and the need for ongoing expert treatments. Similarly, children with progressive neuromuscular disorders may be supported with long-term mechanical ventilation, extending life while potentially increasing suffering and limiting experiential goods such as mobility, communication and autonomy [3,5,10]. In such cases, clinicians must weigh potential benefit against potential harm, yet these concepts resist purely clinical definition as each medical case could have a different presentation and outcome. In each case, clinicians must determine whether continued intervention serves the child’s best interests or merely prolongs their suffering and dying process.

One of the main issues lies in the fact that benefit and harm may have different perceptions and meanings depending on the family’s mentality, level of education, social and economic background, and even more, it depends on religious and cultural beliefs. Benefit may mean survival, neurological function, comfort, or the opportunity for meaningful relationships. Harm may include physical pain, psychological distress, loss of autonomy, independence and self-determination, or even prolonged dying. Thus, both families and clinicians may prioritise these dimensions differently, leading to ethically and emotionally charged arguments when trying to decide in the patient’s best interest. These tensions and disagreements are also amplified by the prognostic uncertainty of each clinical case. Another problem is that PICU clinicians often must make critical decisions before the outcomes become clear, relying on incomplete data and population-level statistics that may not capture the individual trajectories, the personal possibilities and the distinctive features of their own patient. As a result, interventions that initially appear proportionate may later be reinterpreted as harmful when anticipated benefits fail to come to life for each individual. The ethical challenge then shifts from whether to initiate treatment to whether to continue it if it constitutes harm when things do not go the desired way, and improvement fails to materialise. Under these circumstances, without legal clarity or institutional support, clinicians may hesitate to initiate discussions about treatment limitation, fearing conflict with families or legal consequences.

Therefore, moral distress frequently arises when clinicians believe that ongoing treatment no longer serves the child’s best interests, but they feel unable to recommend limitation due to parental insistence, institutional or social culture or even fear of legal consequences [5,10]. This distress is not merely emotional but moral, reflecting a perceived failure to act in accordance with one’s professional and ethical commitments.

In Greece, these challenges and ethical judgements are intensified and complicated by the absence of clear legal frameworks governing the withholding or withdrawal policies of life-sustaining treatments. Clinicians, especially those who work in emergency settings, have to act without formal guidance and a legal framework, and they need to continue applying aggressive interventions as a form of defensive medicine. This approach, while legally cautious, may conflict with ethical obligations to avoid harm and respect each patient’s proportionality, dignity, personality and desires. Over time, such practices may risk normalising the prolongation of suffering and eroding the clinicians’ moral integrity.

While the Greek law recognises the principle of human dignity under Article 2 of the Constitution, it does not clearly articulate how this principle should be applied in end-of-life care. Withdrawal of life-sustaining treatment is often perceived - incorrectly but pervasively - as legally risky, potentially exposing clinicians to criminal liability under provisions related to homicide or bodily harm. As a result, clinicians may feel obliged to continue aggressive treatment even when they believe it no longer offers meaningful benefit to the patient. This defensive approach prioritises one’s legal self-protection over ethical proportionality, potentially leading to prolonged suffering for the child, excessive emotional distress and agony for the grieving family and moral distress and anxiety for the healthcare professionals. Moral distress also arises when clinicians feel unable to act in accordance with their ethical judgment due to external constraints, including legal ambiguity and institutional and cultural morals [5,10].

Addressing beneficence and non-maleficence in the PICU, therefore, requires institutional mechanisms that support ethical deliberation. Structured approaches to beneficence and non-maleficence are therefore essential. Some points of consideration should be the early goals-of-care discussions, framed around proportionality and the child’s best interest, that could potentially help align expectations and prevent escalation to non-beneficial treatment. Ethics consultation services (these are largely absent in many Greek hospitals) could provide critical support by facilitating interdisciplinary deliberation, mediating conflicts between clinicians and families and documenting ethical reasoning.

In addition to the above and beyond formal mechanisms, fostering a culture of reflective practice within PICU teams is considered crucial. These could include regular debriefings following ethically challenging cases on top of formal ethics education that can help clinicians process moral distress, acknowledge uncertainty, learn from experience and sustain moral integrity. In the absence of such support, prolonged exposure to ethically distressing situations may contribute to physical and psychological burnout, emotional detachment and could result in deep erosion of professional values, undermining both personal well-being and quality of care.

Justice

Justice in paediatric intensive care extends beyond bedside decision-making to encompass ethically crucial global questions such as fairness, dignity, self-respect, equity and societal responsibility [9]. PICU is a resource-intensive environment, dependent on highly specialised staff, advanced technology, expensive equipment and limited physical capacity. Scarcity is not an abstract concern but a daily reality, particularly during periods of increased demand, particularly in publicly funded healthcare systems that are constantly facing economic constraints.

Justice in the PICU context concerns the fair and equitable allocation of limited resources, including beds, staff, highly technological equipment and advanced therapies, such as ECMO. Allocation of resource-intensive interventions may favour one patient over another, highlighting the ethical tension between individual benefit and fairness [9].

Consider a scenario in which two critically ill children simultaneously require invasive ventilation and only one ICU bed is available, or even worse, they are in critical need of an advanced therapy, such as ECMO support, but only one circuit and team are available. Let’s complicate things further by adding some clinical information in this unpleasant scenario. Let’s assume that one child has acute, potentially reversible respiratory failure; the other has multi-organ failure in the context of advanced malignancy. Choosing between them requires clinicians to weigh prognosis, likelihood of benefit and ethical obligations to fairness in order to allocate the available medical resources the best possible way. Such decisions are emotionally devastating and morally weighty, as they involve prioritising one life over another; considering that this could involve young children, the procedure and the situation could be described as cruel and pernicious [9].

Justice also arises in more subtle ways, such as prolonged occupation of PICU beds by patients with minimal chances of recovery, potentially delaying admission and limiting access for others who might benefit more from ICU care. These situations force clinicians to confront the tension between individual defence and distributive justice. While clinicians are trained to advocate fiercely for their individual patients, the PICU context demands consideration of broader population-level involvement and balanced decisions [9]. This tension is particularly acute in resource-constrained systems, where every bed and intervention carries opportunity costs and loss of economic resources.

In the absence of clear institutional policies or access to ethics committees, these decisions about resource allocation often fall to individual clinician judgment or teams, leading to variability and potential bias. This could be inconsistent and extremely emotionally draining. Factors such as socioeconomic status, parental assertiveness, personal beliefs and morals or emotional resonance may unconsciously influence doctors’ decisions. Lack of transparency can further erode trust among staff and families, particularly when outcomes are considered unfavourable.

Developing transparent and standardised institutional protocols and ethics consultation services is essential to ensure fairness, reduce moral distress and help to promote public trust. Such protocols should be adaptable, transparent, and supported by ethics committees that provide guidance during crises. Importantly, justice in the PICU also requires attention to systemic inequities, including disparities in access to care and outcomes across populations [1,9]. Ethical decision-making about justice cannot be isolated from societal values. The way scarce resources are allocated usually reflects the collective judgment about whose lives are prioritised and why.

In Greece, the lack of standardised national or institutional allocation protocols means that such decisions often rest on individual clinician judgment. Such dependency on personal discretion increases both the variability and the risk of bias, while also placing an enormous moral burden on clinicians’ shoulders. These decisions, made without transparent criteria or without the review and the formal opinion of an ethics committee, may appear arbitrary to families and staff, undermining trust and leading to disagreement and conflict. Therefore, developing transparent, ethically grounded institutional policies for guiding resource allocation is considered absolutely essential. Such policies should be adaptable, publicly defensible, within legal frameworks and supported by ethics committees that provide guidance during times of crises. Justice in the PICU also requires attention to systemic inequities, including disparities in access to care, regional differences in PICU capacity and socioeconomic factors influencing patients’ outcomes [1,9]. Ultimately, justice in the paediatric intensive care setting cannot be separated from the values that govern each society. The ways in which scarce resources are allocated reflect collective judgments about vulnerability, worth and responsibility. Ensuring that these judgments are ethically defensible and publicly accountable is crucial for maintaining trust in paediatric critical care institutions and in the healthcare system in general. 

Gaps in bioethics training and institutional support in PICU settings

Structured bioethics education for healthcare professionals working in PICUs in Greece remains limited and inconsistently integrated into medical and nursing training programs [5,6,10]. As a result, many clinicians are required to confront highly complex ethical dilemmas in the absence of formal preparation, standardised guidance, or institutional support mechanisms. Decisions concerning limitation or withdrawal of life-sustaining treatment, end-of-life care, prognostic uncertainty, parental disagreement, and assessments of the child’s best interests demand not only advanced clinical expertise but also well-developed ethical reasoning, communication skills, and emotional competence. In practice, however, these competencies are often acquired informally through clinical experience rather than through structured educational frameworks.

The absence of systematic ethics training may contribute to variability in clinical decision-making and increase the emotional burden placed on healthcare professionals. Under conditions of time pressure, uncertainty, and emotional strain, clinicians frequently rely on personal values, prior experiences, and informal collegial support when addressing ethically challenging situations. While experiential learning remains important, the lack of consistent ethical frameworks may lead to inconsistencies in practice and intensify moral distress among both physicians and nurses. Existing educational curricula often provide limited exposure to key domains such as ethical decision-making, conflict resolution, end-of-life communication, shared decision-making, and discussions with families during situations perceived as medically futile or emotionally overwhelming.

These challenges are further compounded by the limited availability of institutional ethics support structures within many PICU settings. Multidisciplinary ethics committees, formal ethics consultation services, and written institutional guidelines are not routinely accessible in several healthcare environments. Consequently, difficult ethical decisions are frequently managed through informal team discussions or individual moral judgment. Although collegial deliberation may strengthen team cohesion, reliance on informal processes alone can leave clinicians vulnerable to uncertainty, professional isolation, and cumulative moral stress. Over time, repeated exposure to ethically distressing situations without adequate institutional support may negatively affect professional resilience, contribute to burnout, and influence clinicians’ future interactions with children and families during periods of profound vulnerability.

International experience demonstrates that systematic bioethics education can substantially enhance ethical competence in pediatric critical care practice. Educational interventions incorporating case-based learning, reflective practice, simulation-based communication training, multidisciplinary discussion, and structured ethics consultation have been associated with improvements in ethical reasoning, decision-making confidence, and communication with families. Such approaches promote more consistent, transparent, and patient- and family-centred care while supporting collaborative clinical decision-making. International organisations, including the World Health Organisation and the Council of Europe, have emphasised that ethical competence should be regarded as a core clinical skill rather than a purely theoretical or optional component of professional education. Accordingly, the integration of formal bioethics training into undergraduate medical education, postgraduate specialty training, and continuing professional development represents an important priority for contemporary pediatric critical care practice [5,10].

Importantly, strengthening ethical practice in PICUs extends beyond education alone and requires the development of supportive institutional cultures. Ethical decision-making is more sustainable when clinicians are encouraged to express uncertainty, engage openly in interdisciplinary dialogue, and acknowledge the emotional impact of caring for critically ill children. The establishment of protected spaces for reflective discussion, routine access to ethics consultation services, and psychological support following morally distressing cases may foster a more supportive and humane clinical environment. Such measures recognise that ethical challenges in pediatric intensive care are not isolated theoretical problems but continuous and deeply relational processes involving clinicians, children, and families confronting uncertainty, suffering, and difficult decisions.

Addressing current gaps in bioethics training and institutional support, therefore, represents both an educational and organisational priority. Investment in structured ethics education, accessible consultation pathways, and multidisciplinary support systems may better equip PICU clinicians to navigate ethically complex situations with greater clarity, consistency, professionalism, and compassion, while simultaneously reducing the personal burden associated with moral distress and ethically challenging care.

Implementation, evaluation, and practical steps for adoption

Implementation of a structured ethics education programme in the PICU should be approached as a pragmatic service-development initiative embedded within existing clinical governance and teaching structures. Initial steps include securing the alignment between PICU leadership and the institutional ethics service, appointing a clinical education lead and ethics liaison and adopting a standardised case-analysis framework to ensure consistency across teaching activities. To maximise feasibility, core activities (e.g., multidisciplinary ethics rounds and brief post-case debriefs) should be integrated into established meeting rhythms and oriented explicitly toward reflective, non-punitive learning, with attention to psychological safety and team cohesion. Evaluation should combine process indicators (participation rates, frequency of rounds/debriefs, and patterns and timing of ethics consultation relative to the onset of conflict) with outcome indicators that capture ethically relevant practice change [11,12]. These may include staff ethical self-efficacy and preparedness for difficult conversations, measures of moral distress where validated instruments are available, and practice-based markers such as the clarity, completeness, and longitudinal coherence of goals-of-care documentation and decision rationales across shifts and disciplines. Where feasible, structured family feedback on communication quality and perceived inclusion may provide an additional perspective on whether the programme improves transparency and trust in decision-making processes [13-15].

In parallel, near-term adoption can be supported through a focused set of low-burden interventions that operationalise ethical principles in everyday care. PICUs may begin by instituting recurring multidisciplinary ethics rounds grounded in real cases and guided by a consistent template (clinical facts, decision points, values/principles in tension, justification, and agreed next steps), complemented by brief post-case ethics debriefs following ethically challenging events to consolidate learning and identify procedural improvements. Establishing locally agreed “trigger” criteria for early ethics involvement can reduce unwarranted variation in practice and prevent escalation of disagreements by ensuring timely support for families and staff. Standardising documentation prompts for ethically complex decisions (explicit goals of care, benefit-burden rationale, areas of disagreement, agreed plan, and review points) further promotes continuity and accountability. Collectively, these steps strengthen the translation of ethical principles into durable clinical practice by combining education, supportive processes, and documentation standards that are achievable within the constraints of the PICU environment [1,14-17,18,19,20].

Discussion

The application of the four bioethical principles in paediatric intensive care demands deliberative judgment grounded in situational awareness and recognising the complexities of each situation. Autonomy, beneficence, non-maleficence and justice are four fundamental principles that are absolutely interconnected, requiring clinicians to balance these principles dynamically in each district clinical case rather than applying them in isolation. Respecting autonomy may conflict with beneficence when adolescents refuse potentially life-saving treatment, while justice may be challenged by resource scarcity in eras of increased demand for resources. These dilemmas highlight the need for reflective, structured and human-centred ethical reasoning in practice [1,2,3].

In Greece, PICUs face additional structural challenges, including limited legal guidance on treatment limitation, scarcity of ethics committees that should be present in hospitals or institutions and minimal structured ethics education at both the under- and post-graduate levels. Not to mention the underdeveloped existing legislation regarding end-of-life care, emphasising the urgent need for both educational and systemic support and reflective individual practice [5,10]. Ethical decision-making in PICUs extends beyond procedural adherence; it involves understanding of the child’s experiences, the family’s values and beliefs, along with the broader socio-economic context.

Structured ethics education, including case-based discussions, simulation exercises, and access to ethics committees, can enhance clinicians’ ability to navigate complex and challenging ethical scenarios. The solution of adapting the international guidelines to the local context ensures that ethical frameworks are practical and actionable. Most importantly, providing psychological support acknowledges the emotional realities of the profound challenges that clinicians face every day in the ICU environment, promoting sustainable, high-quality care, equity and transparency. Ultimately, ethical competence in paediatric intensive care combines formal education, reflective practice and a human-centred approach that integrates clinical expertise, ethical principles, including always the perspectives of patients and families, aligning medical interventions with the best interests of the child. [1,2,5,6,10,12,17-19,21]. A proposed structured approach for this educational model in implementing ethics education in PICU is shown in box 1. In Table 1, the four fundamental principles are described along with examples and the emerging need for education and support.

Box 1: Proposed Structured Ethics Education Model for the PICU

Aim: To strengthen ethically consistent, defensible decision-making in the PICU by embedding practical ethics education into routine clinical work and equipping multidisciplinary teams with applied reasoning, communication, and documentation skills.

The core components (embedded in the clinical workflow) are as follows: (1) Case-based ethics rounds (multidisciplinary) - regular discussion of real PICU cases using a consistent analytic framework to build shared language and promote coherent ethical reasoning across professions. (2) Brief post-case ethics debriefs - short, structured debriefs following ethically challenging cases to consolidate learning, surface moral distress, and identify process improvements. (3) Simulation/role-play for high-stakes conversations - skills-based training focused on recurrent PICU discussions (e.g., limitation of life-sustaining treatment, disagreement about goals of care, communication of uncertainty), with feedback to translate ethical analysis into communicative competence. (4) Microlearning modules aligned with recurring PICU dilemmas - short, repeatable teaching units to reinforce key concepts longitudinally and support consistent baseline competence among rotating staff. (5) Ethics “trigger” criteria and early ethics involvement - locally agreed prompts for timely ethics support (e.g., persistent disagreement, prolonged invasive support with low expected benefit, repeated escalation requests despite substantial burdens, significant team moral distress), aimed at preventing avoidable escalation and reducing unwarranted variation in practice.

Competency targets (what the programme should teach) include best-interests reasoning and its practical application in paediatric critical care; proportionality and benefit-burden assessment under prognostic uncertainty (integrating beneficence and non-maleficence); parental authority and its limits, including the child’s developing interests and, where appropriate, assent; justice considerations relevant to intensive care practice and institutional processes; management of intra-team disagreement and recognition/mitigation of moral distress; and transparent documentation of ethical reasoning (goals of care, options considered, rationale, areas of disagreement, agreed plan, and review points).

Implementation features (to support feasibility and sustainability) include multidisciplinary participation with a designated PICU education lead and ethics liaison; use of a standard case-analysis template to ensure consistency across sessions; integration into existing educational structures (e.g., departmental teaching, multidisciplinary meetings); and explicit commitment to psychological safety and a non-punitive reflective culture.

Evaluation indicators (to assess impact) include process measures (participation, frequency of rounds/debriefs, timing and use of ethics consultation) and outcomes (staff ethical self-efficacy, moral distress (where feasible), quality/consistency of goals-of-care documentation, and (where feasible) family-reported communication and inclusion in decision-making).

The main findings of this review are shown in Table 1.

**Table 1:** Four fundamental principles, with challenges, examples, and the detailed need for education and support.

Bioethical principle	Challenges in the PICU	Clinical examples	Need for education and support
Autonomy (respecting patient/family decision-making)	Children often cannot provide informed consent. Parents’ decisions may conflict with medical recommendations. Cultural or religious beliefs influencing decisions.	Parents refuse life-sustaining treatment due to religious reasons. Adolescents wishing to participate in care decisions.	Training clinicians in age-appropriate communication. Role-playing scenarios for shared decision-making. Education on cultural competence and legal frameworks.
Beneficence (promoting the patient’s best interests)	Determining best interest in complex cases. Conflicting opinions among healthcare team members. Limited resources influencing care options.	Deciding on aggressive intervention for critically ill neonates with poor prognosis.	Case-based learning on balancing clinical benefit vs. burden. Ethics seminars on emerging treatments and outcomes. Guidance on risk-benefit assessment.
Non-maleficence (do no harm)	High-risk interventions may unintentionally cause harm- Uncertainty in prognostic outcomes. Emotional stress affecting clinical judgment.	Administering experimental therapies with potential severe side effects. Repeated painful procedures on fragile patients.	Workshops on procedural ethics and risk minimisation. Simulation-based training for complex procedures. Debriefing sessions for psychological resilience.
Justice (fair allocation of resources)	Scarce ICU resources during crises. Inequities in access to care. Ethical dilemmas in prioritisation of patients.	Choosing which patient receives a ventilator during peak demand. Disparities in access for children from different socioeconomic backgrounds.	Education on resource allocation frameworks. Interdisciplinary ethics discussions and committee involvement. Policy training and awareness of institutional guidelines.

Limitations

This narrative review may introduce selection bias due to the nature of the included literature. Furthermore, empirical data on practical ethics education and practice in Greek PICUs remain limited and lacking. This underlines and supports greatly the necessity to organise rigorously structured, large-scale prospective studies in order to evaluate the impact of clearly articulated ethics programs in the clinical settings. Very crucial remains also the future presence of ethics committees in the hospitals, the specific legal context that would provide guidance to clinicians and the institutional support for patient outcomes. Last but not the least, one should not forget the impact and value of the clinicians’ well-being and resilience when they are properly supported and respected in the professional environment.

## Conclusions

This review synthesises the current literature on ethical practice in PICUs, identifies common challenges, and explores feasible future strategies to strengthen the ethics education and the institutional support that needs immediate implementation in the Greek context.

The four bioethical principles - autonomy, beneficence, non-maleficence, and justice - are fundamental to ethical practice in the paediatric intensive care setting. However, their meaningful implementation in everyday clinical settings requires more than theoretical awareness. Their effective application depends on structured ethics education, experiential training and robust institutional support, including ethics committees inside the hospitals, a precise legal framework and interdisciplinary collaboration. Simulation-based learning, case discussions and interdisciplinary ethics workshops offer practical opportunities to engage with real-world challenges, fostering reflective practice and professional growth, personal self-esteem in decision making and team resilience.

The necessity of adapting international guidelines to the Greek healthcare context, alongside substantial psychological support for clinicians, is essential for navigating high-stakes decisions ethically and sustainably, with confidence and consistency. It is strongly believed that by implementing these measures, the ethical competence will be enhanced, the patient care will be strengthened, and it will ensure that PICU decisions will prioritise the best interests of children while maintaining equity, transparency and strong public trust. Investment in structured ethics education - beginning in undergraduate medical training and extending through continuing professional development - can enhance clinicians’ ethical reasoning skills, improve communication with families and promote shared decision-making in this profoundly challenging setting.
